# The Kinetic Signature of Toxicity of Four Heavy Metals and Their Mixtures on MCF7 Breast Cancer Cell Line ^†^

**DOI:** 10.3390/ijerph10105209

**Published:** 2013-10-21

**Authors:** Egbe Egiebor, Adam Tulu, Nadia Abou-Zeid, Isoken Tito Aighewi, Ali Ishaque

**Affiliations:** 1 Department of Natural Sciences, University of Maryland Eastern Shore, Princess Anne, MD 21853, USA; E-Mails: honeyspringsbv@yahoo.com (E.E.); adamtulu@yahoo.com (A.T.); nzeid06@yahoo.com (N.A.-Z.); 2 Department of Biology, Chemistry and Environmental Health Sciences, Benedict College, Columbia, SC 29204, USA; E-Mail: aighewii@benedict.edu

**Keywords:** heavy metals, real time electronic cell sensing, glutathione, L-buthionine sulphoximine

## Abstract

This study evaluated the kinetic signature of toxicity of four heavy metals known to cause severe health and environmental issues—cadmium (Cd), mercury (Hg) lead (Pb) arsenic (As)—and the mixture of all four metals (Mix) on MCF7 cancer cells, in the presence and absence of the antioxidant glutathione (GSH). The study was carried out using real time cell electronic sensing (RT-CES). RT-CES monitors in real time the electrical impedance changes at the electrode/culture medium interface due to the number of adhered cells, which is used as an index of cell viability. Cells were seeded for 24 h before exposure to the metals and their mixtures. The results showed that in the presence and absence of cellular glutathione, arsenic was the most cytotoxic of all five treatments, inducing cell death after 5 h of exposure. Lead was the least cytotoxic in both scenarios. In the presence of cellular GSH, the cytotoxic trend was As > Cd > MIX > Hg > Pb, while in the absence of GSH, the cytotoxic trend was As > Hg > MIX > Cd > Pb. The findings from this study indicate the significance of glutathione-mediated toxicity of the metals examined—particularly for mercury—and may be clinically relevant for disorders such as autism spectrum disorder where decreased glutathione-based detoxification capacity is associated with increased mercury intoxication.

## 1. Introduction

Among heavy metals, cadmium (Cd), mercury (Hg), lead (Pb) and arsenic (As) are some of the most toxic elements due to their persistence in the environment. They cause oxidative and nitrosative stress [1,2,3] and damage macromolecules in cells which consequently lead to cell death by apoptosis or necrosis [4]. Although the toxicity of individual heavy metals on organisms are of serious ecological consequences, metals rarely occur alone, most often occuring as mixtures [5]. Metal mixtures substantially complicate the risk assessment process for these elements and very few researchers have studied the environmental effects of mixtures of heavy metals. Information on potential adverse health effects associated with intake of mixture of most frequently found metals contaminant is not known. Hence, it is imperative to study heavy metals not only individually, but also in mixtures.

Metal metabolism has significant effects on metal toxicity. Protective processes at the molecular and cellular levels may not affect cellular homeostasis even after metal exposure. A studied example that is known to modify the susceptibility to metals is glutathione (GSH) [6]. GSH can interfere with toxic metals by altering the rates of metal uptake and elimination [7] and it can protect against oxidative stress resulting from metal-catalysed redox reactions [8].

The goal of this study was to understand the kinetic signature of toxicity of four heavy metals (As, Cd, Hg and Pb) and the mixture of all four metals (Mix) on MCF7 cells, in the presence and absence of cellular glutathione, using real time cell electronic sensing (RTCES). The present study highlights the viability of MCF7 cells that were exposed to metals and their mixture in real time. To study the effect of the absence of GSH in the cell, cellular GSH synthesis was inhibited by exposing the cells to l-buthionine sulfoximine (LBSO) for 24 h. LBSO is a specific γ-glutamylcysteine synthetase inhibitor that irreversibly inhibits the rate-limiting step of glutathionine biosynthesis and depletes the intracellular GSH pool in both cultured cells and in whole animals.

## 2. Materials and Methods

### 2.1. Chemicals

Atomic absorption standards (Acros Organics, NJ, USA) consisted of arsenic 1 mg/L in 2% KOH, cadmium 1 mg/L in 0.5 N nitric acid, lead 1 mg/L in 2% nitric acid and mercury 1 mg/L in 10% nitric acid. L-Buthionine sulfoximine (LBSO) was purchased from Toronto Research Chemicals (North York, ON, Canada).

### 2.2. Cell Lines and Culturing Reagent

MCF7 cell lines were purchased from American Type Culture Collection (ATTC, Manassas, VA, USA). Minimum Essential Medium (MEM) alpha 1x, Dulbecco’s Phosphate Buffered Saline (PBS), MEM without phenol, and penicillin streptomycin were purchased from GIBCO Invitrogen (Grand Island, NY, USA). Trypsin-EDTA and fetal bovine serum (FBS) were purchased from ATTC. MCF7 cells were grown in MEM alpha 1×supplemented with 10.0% FBS and 1.0% penicillin streptomycin. The cells were grown in a humidified incubator kept at the temperature of 37 °C and 5% carbon dioxide.

### 2.3. Description of Exposure Studies

#### 2.3.1. Real-Time Cell Electronic Sensing (RT-CES)

The *in vitro* cell viability effect of individual and mixture with/without LBSO on MCF7 cells were determined by RT-CES cytotoxicity assay. Cells in the presence of individual metals at concentrations from 0 µg/mL to 21.7 µg/mL were monitored by measurements of electrical impedance (ACEA Biosciences Inc., San Diego, CA, USA) every 10 min for 96 h. Continuous recording of impedance in cells was reflected by cell index value [9].

#### 2.3.2. Testing the Kinetic Response of the Individual Metals on MCF7 Cells

To determine the individual toxicity of the metals, MCF7 cells were seeded in a 16x E-plate device and grown in the incubator for 24 h for metal treatment. To create a negative control, the last row of cell culture plate contained the media and cells but was not exposed to any metal. After 24 h, the media in the seeded cells was dumped, 180 µL of fresh media was added to each well and 50 µL of serially diluted metals (concentrations ranging from 0 µg/mL–21.7 µg/mL) was also added to give a final volume of 230 µL. Using four seeded plates (one for each metal), The first row of the plates had the highest concentration of the individual metals and concentrations of As, Cd, Hg, and Pb decreased from row 1 to row 7. Row 8 was not treated with any metals. The cells were incubated for 96 h. The procedure was carried out in duplicates and repeated twice for each of the chemicals to make sure the trend of toxicity was similar.

#### 2.3.3. Testing the Kinetic Response of Quaternary Mixture of Metals on MCF7 Cells

A mixture of the four metals was made by mixing As, Cd, Hg, and Pb stock solutions in the ratio of their Environmental Protection Agency (EPA) Maximum Contaminant Level (MCL), that is 10, 5, 2 and 15 ppb, respectively. A serial dilution of the mixture was made such that the starting concentrations for As, Cd, Hg, and Pb in mixture were 250, 125, 50 and 375 mg/L, respectively. MCF7 cells were seeded in a 16x E-plates for 24 h and were treated with decreasing concentration of the mixture as mentioned above. The treated cells were incubated for 96 h. The procedure was carried out in duplicates and repeated twice.

#### 2.3.4. Testing the Kinetic Response of Individual and Mixture of Metals on LBSO Pretreated MCF7 Cells

To determine the toxicity of the individual and composite mixture of metals in the absence of glutathione (GSH), 2.5 mM of GSH-depleting agent LBSO was used to seed the cells prior to exposure to the chemicals. The appropriate concentration of which did not kill more than 5% of the cells was predetermined to be 2.5 mM in prior experiments. MCF7 cells were seeded and incubated for 24 h using the growth medium containing 2.5 mM LBSO. Thereafter, each plate was treated with decreasing concentrations of the four metals as described previously. The treated cells were incubated for 96 h.

## 3. Results

### 3.1. Kinetic Response of Individual and Composite Mixture of Metals on MCF7 Cells

To characterize the kinetic signature of each of the four chemicals (Cd, Hg, Pb and As) and the mixture of all four (Mix), MCF7 cells were exposed to different concentrations of each chemical and dynamically monitored over 96 h using real time cell electronic sensing (RT-CES). RT-CES measures cell viability in real time using electrical impedance [9]. Using the aforementioned methods, the four highly toxic chemicals and their mixture were found to be cytotoxic within the concentration range that was tested. The kinetics response of MCF7 was different for each chemical.

**Figure 1:**
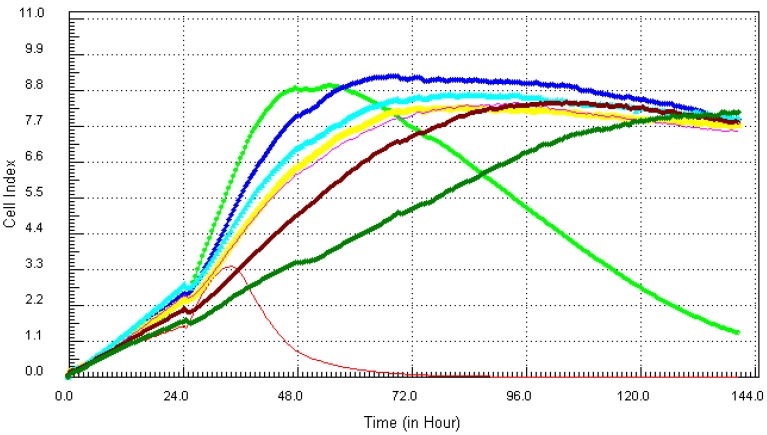
Kinetics of cytotoxic responses for Cadmium (Cd) in MCF7 cells monitored by the RT-CES system.10,000 cells/well were plated in 16-well strips for the RT-CES cytotoxicity assay. Different compound concentrations in µg/mL are indicated by different colors. Red = 21.7, light green = 10.8, blue = 5.4, yellow = 2.7, turquoise = 1.3, pink = 0.68, burgundy = 0.34, green = control.

**Figure 2:**
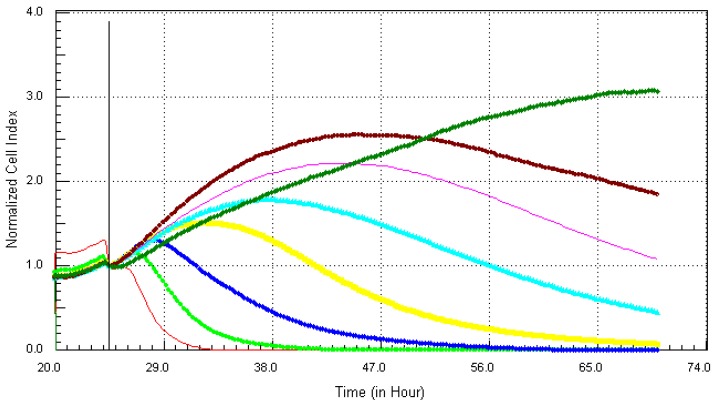
Kinetics of cytotoxic responses for arsenic (As) in MCF7 cells as described in Figure 1 legend.

Cadmium, one of the more notorious chemicals, of serious concern due to its ability to cause lung and prostate cancer, was cytotoxic after 12 h at the highest concentration of 21.7 µg/mL (red, Figure 1). At the concentration of 10.8 µg/mL (light green), cadmium induced cell death after 34 h of exposure to MCF7 cells. Lower concentrations of cadmium ranging from 0.34 ppm to 5.4 ppm did not show any visible effect on the cells. Monitoring the kinetic signature of each chemical on MCF7 cells in real time, the results showed that arsenic which is known to be associated with different types of cancers, cardiovascular diseases and neurological disorders [10], had the worst effects on the cells (Figure 2).

**Figure 3:**
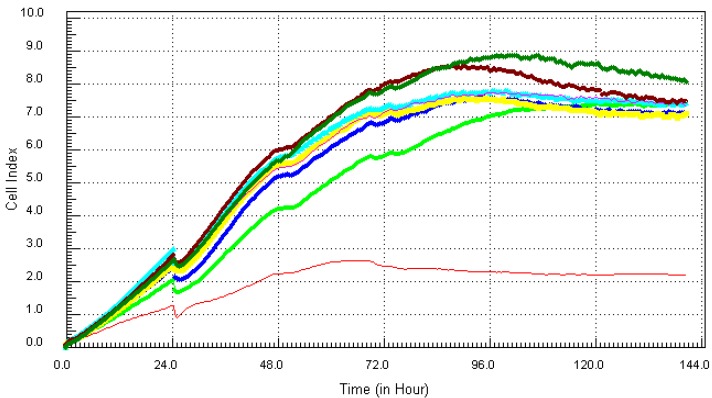
Kinetics of cytotoxic responses for mercury (Hg) in MCF7 cells as described in Figure 1 legend.

**Figure 4:**
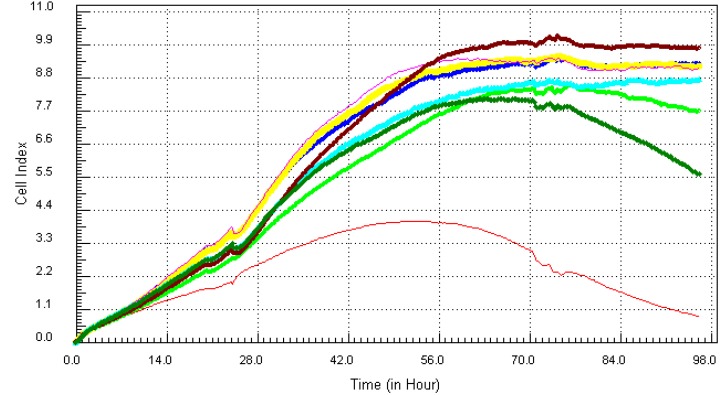
Kinetics of cytotoxic responses for lead (Pb) in MCF7 cells as described in Figure 1 legend.

The highest concentration of 21.7 µg/mL (red) induced cytotoxic effects after just 2 h of exposure and induced total cell death after 5 h. At concentrations of 10.8 µg/mL (light green) and 5.4 µg/mL (blue), arsenic induced total cell death at 10 h and 20 h after exposure respectively. Lower concentrations of arsenic ranging 0.34 ppm to 2.7 µg/mL showed slower onset of cytotoxicity but induced obvious cell death. Mercury which has been linked with various types of tissue damages [11], showed no visible effect on cell viability (Figure 3). However,the highest concentration of mercury 21.7 µg/mL (red) inhibited cell proliferation but did not cause any cell death or decrease in cell number. From our results (Figure 4), lead, which is known for its neurotoxic effects, showed cytotoxic effects at the highest concentration of 21.7 µg/mL (red) after about 34 h of exposure but all other concentrations did not induce any visible cell death.

**Figure 5:**
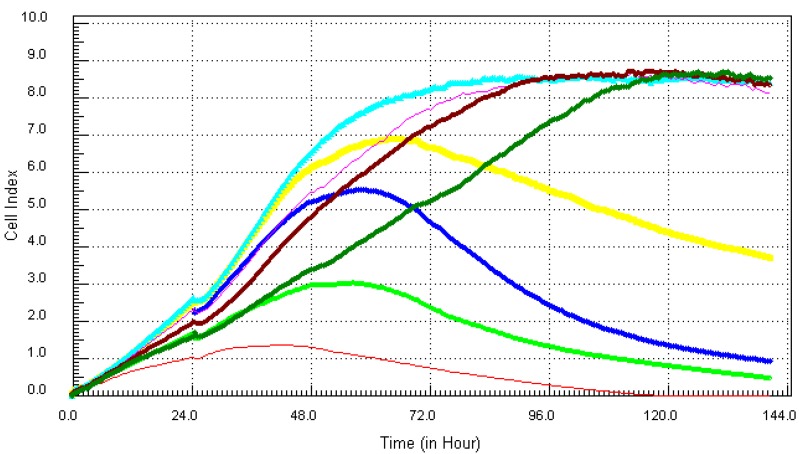
Kinetics of cytotoxic responses for mixture (Mix) in MCF7 as described in Figure 1 legend.

This study also examined the effects of the combination of all four chemicals on MCF7 cell lines (Figure 5). Though there was a slow onset of cytotoxicity, the four higher concentrations of the mixture induced cell death after 72 h of exposure. The three lower concentrations of the mixture did not have any visible effect on cell viability.

### 3.2. Kinetic Response of Individual and Quaternary Mixture of Metals on LBSO Pretreated MCF7 Cells

To characterize the kinetic signature of each of the four chemicals (Cd, Hg, Pb and As) and their mixture (Mix) in the absence of cellular glutathione, MCF7 cells was pretreated with LBSO (a compound that irreversibly inhibits GSH synthesis) for 24 h and the cells were exposed to different concentrations of each chemical and their quaternary mixture. The cytotoxic effects of each chemical and their mixture on MCF7 cells were dynamically monitored in real time for 96 h.

**Figure 6:**
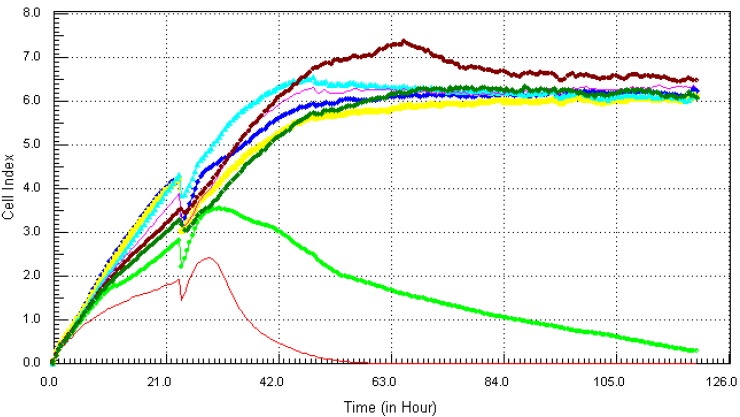
Kinetics of cytotoxic responses for cadmium in LBSO-treated MCF7 cells monitored by the RT-CES system. 10,000 cells/well were plated in 16-well strips for the RT-CES cytotoxicity assay. Different compound concentrations in µg/mL are indicated by different colors. Red = 21.7, light green = 10.8, blue = 5.4, yellow = 2.7, turquoise = 1.3, pink = 0.68, burgundy = 0.34, green = control.

**Figure 7:**
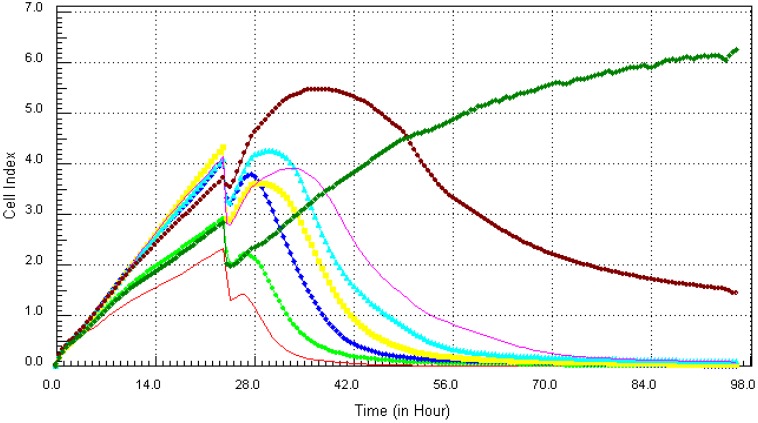
Kinetics of cytotoxic responses for arsenic in LBSO-treated MCF7 cells as described in Figure 6 legend.

**Figure 8:**
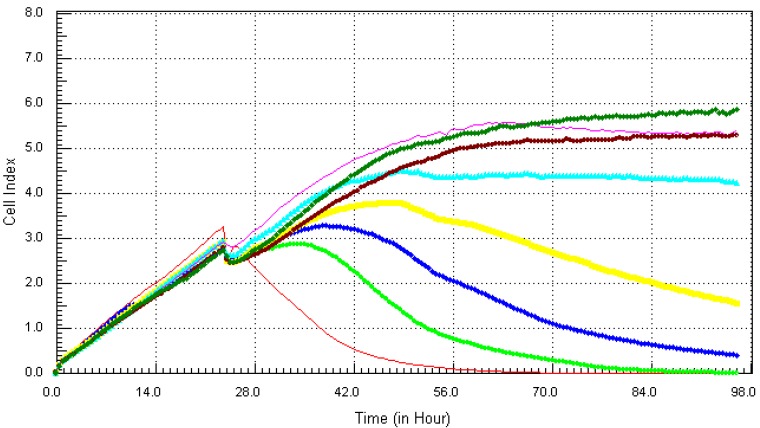
Kinetics of cytotoxic responses for mercury in LBSO-treated MCF7 cells as described in Figure 6 legend.

**Figure 9:**
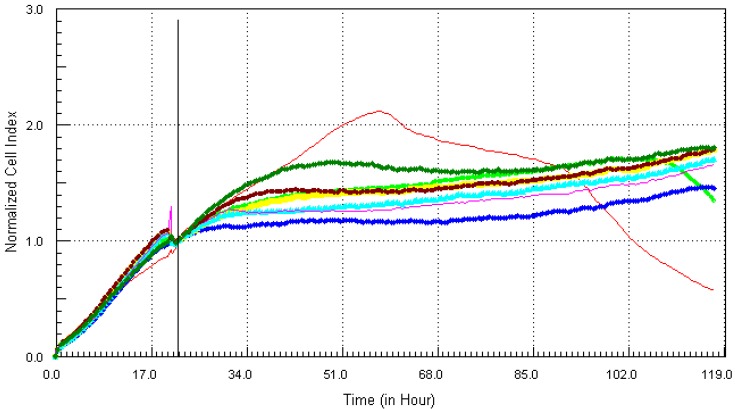
Kinetics of cytotoxicity responses for lead in LBSO-treated MCF7 cells as described in Figure 6 legend.

**Figure 10:**
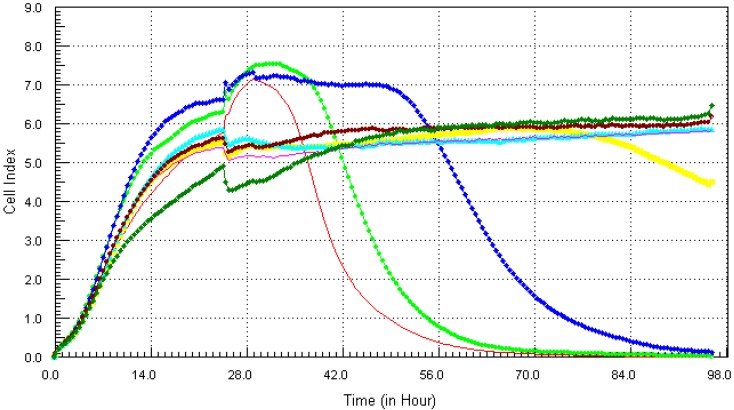
Kinetics of cytotoxicity responses for mixture in LBSO-treated MCF7 cells as described in Figure 6 legend.

Following the method that was described previously, the individual chemicals and their mixture (except lead) were found to be cytotoxic within the concentration range that was tested. The kinetics response of LBSO treated MCF7 cells was different for each chemical. Cadmium (Figure 6), arsenic (Figure 7), mercury (Figure 8) and the mixture of all four chemicals (mix) (Figure 10) induced severe cytotoxic effects on MCF7 cells, while lead (Figure 9) showed a visible decrease in cell number 40 h after exposure to the highest concentration. The result shows that mercury and arsenic had severe cytotoxicity after only 4 h of exposure. All concentrations (0.34 ppm–21.7 ppm) of arsenic were fully cytotoxic after 32 h of exposure. After 32 h of exposure, the four higher concentrations (2.7 µg/mL–21.7 µg/mL) of mercury induced cytotoxic effects while the lower three concentrations (0.034 µg/mL–1.3 µg/mL) did not have any effects on the cells. The higher two concentrations (10.8 ppm–21.7 ppm) of cadmium induced cytotoxic effect after 6 h of exposure, while the lower concentration did not reveal any visible effects on MCF-7 cells. In the case of the mixture of all four chemicals (mix), the higher three concentrations induced cell death after 32 h of exposure, while the lower concentration showed neither a decrease in cell number nor an increase in cell proliferation. The highest concentration (21.7 ppm) of lead showed a little decrease in cell number after 40 h of exposure, but all other concentrations did not show any visible effect on MCF-7 cells.

## 4. Discussion and Conclusions

Exposure of cells to heavy metals may immediately lead to necrosis or apoptosis, but sometimes, cells may undergo series of events like the synthesis of more glutathione molecules that could lead to their survival. Studies by scientists have widely demonstrated that the exposure to heavy metals shares several primary mechanisms of toxicity, including the production of reactive oxygen species, reaction with intracellular thiols and competing with essential metals in biological systems [12,13].

This study, determined the kinetic signature of toxicity of four heavy metals and their mixture (Hg, Cd, As, Pb and Mix) on MCF-7 breast cancer cells in the presence and absence of glutathione. From the results it was obvious that cells that were treated with the glutathione-scavenging agent LBSO before chemical exposure, exhibited more cell death at a shorter time period than cells that were exposed to the metals alone. The results showed that when MCF-7 cells were exposed to only cadmium the highest concentration of 21.7 µg/mL induced full cytoxicity after 12 h of exposure, the next concentration of 10.8 µg/mL exhibited cytotoxic effects after 34 h of exposure and the lower concentrations of 0.34 ppm–5.4 ppm did not reveal any cytotoxic effects. In contrast, when MCF7 cells were exposed to the glutathione-scavenging compound LBSO before being exposed to cadmium, the two higher concentrations (10.8 µg/mL and 21.7 µg/mL) were fully cytotoxic after only 6 h of exposure. Interestingly, arsenic was more cytotoxic on MCF-7 cells than cadmium both in the presence and absence of glutathione. The higher three concentrations of arsenic (5.4–21.7 µg/mL) induced cytotoxic effects after 5 h of exposure and after 48 h, all concentrations were cytotoxic. In contrast, when cellular glutathione was scavenged, the higher three concentrations of arsenic (5.4–21.7 µg/mL) became cytotoxic at only 4 h while all other concentrations induced cytotoxicity after 24 h of exposure. None of the concentrations of mercury had any obvious effects on MCF-7 cells when cellular GSH was present. In contrast, when cells were pretreated with LBSO before exposure to mercury, the result revealed that Hg was cytotoxic at the highest concentration of 21.7 ppm after only 4 h of exposure, while the next three concentrations (2.7–10.8 µg/mL) induced total cytotoxic effects after 32 h of exposure. No concentrations of lead showed any signs of cytotoxicity, but when the cells were treated with LBSO before being exposed to lead, the highest concentration of 21.7 µg/mL indicated a slight decrease in cell number after 40 h of exposure. 

The higher incidence of cell death occurring in the presence of LBSO may be due to the fact that LBSO scavenges cellular glutathione and potentiates the effects of toxic chemicals [14]. Our result is in accordance with the findings of Lee *et al.*, [15], who discovered that there was an up regulation of glutathione in arsenic treated Chinese hamster ovary cells. In addition, research by [16] found that there was an increase in the activities of glutathione when leydig cells were exposed to cadmium. Increased glutathione activities is in cells exposed to heavy metals may be the reason why MCF7 cells exposed to heavy metals alone showed more resistance than cells that were pretreated with LBSO before metal exposure. Glutathione may have helped the cells fight against reactive oxygen species that exposure to the metals may have generated, hence, the reduced cell death when glutathione was not scavenged. The tripeptide (glutathione) scavenges hydroxyl radicals, detoxifies hydrogen peroxides and lipid hydroperoxides by the activity of glutathione peroxidase and is able to regenerate other antioxidant molecules [17]. Glutathione can also bind with metals like Hg to form the GSH-metal complex which is then pumped out of the cells [14]. Studies has also shown an increase in GSH when NKR-52E cells were treated with Hg and Pb but treatment with Hg depleted more GSH and induces more oxidative stress than Pb because Hg had more affinity for GSH [18]. This may be why our result showed more cell death when glutathione was scavenged from the cells before exposure to mercury than when the cells were exposed to mercury alone. The mercury treated cells may have been able to survive because the metals may have been bound to glutathione molecules and pumped out of the cells to prevent cell death. On the other hand, when glutathione was absent from the cells, mercury had a drastic effect on the cells causing almost immediate cell death. The observation of glutathione's importance in protecting against mercury induced toxicity may be of particular importance for clinical disorders such as autism spectrum disorders. Previous studies by [19] on subjects diagnosed with an autism spectrum disorder revealed that they had decreased glutathione-based detoxification capacity, particularly of mercury, and as a consequence had elevated mercury body-burden. The observations from the present study provide cellular support for how impaired glutathione-based detoxification in autism spectrum disorders may result in mercury intoxication. In the case of the mixture of the four chemicals, the higher three concentrations inhibited cell viability when cellular GSH was intact, but when GSH was scavenged, the mixture induced cytotoxic effects less than 10 h after exposure. The result showed that the onset of cell death in cells exposed to the mixture was sooner in cells treated with LBSO than in those that were not treated with LBSO. From our results, the cytotoxic effects of the metals on MCF7 cells in the presence of glutathione is as follows As > Cd > MIX > Hg > Pb whereas when glutathione is scavenged, the cytotoxic effects of the metals is as follows As > Hg > MIX > Cd > Pb.

Cells that were treated with LBSO before being exposed to the chemicals may have experienced more cell death because of the absence of glutathione and the cells may not have had the ability to fight off ROS generation which in turn leads to cell death. Although cells that were exposed only to the chemicals showed cell death, but it took a longer time and only occurred at high chemical concentrations.

## References

[1] Stohs S.J., Bagchi D. (1995). Oxidative mechanisms in the toxicity of heavy metals. Free. Radical Biol. Med..

[2] Ercal N., Gurer-Orhan H., Aykin-Burns N. (2001). Toxic metals and oxidative stress part I: Mechanisms involved in metal induced oxidative damage. Curr. Top. Med. Chem..

[3] Pompella A., Visvikis A., Paolicchi A., de Tata V., Casini A.F. (2003). The changing faces of glutathione, a cellular protagonist. Biochem. Pharmacol..

[4] Pulido M.D., Parrish A.R. (2003). Metal-induced apoptosis: Mechanisms. Mutat. Res..

[5] Ishaque A., Johnson L., Gerald T., Boucaud D., Okoh J., Tchounwou P. (2006). Assessment of Individual and Combined Toxicities of Four Non-Essential Metals (As, Cd, Hg and Pb) in the Microtox Assay. Int. J. Environ. Res. Public Health..

[6] Kang Y.J., Enger M.D. (1987). Effect of cellular glutathione depletion on cadmium-induced cytotoxicity in human lung carcinoma cells. Cell Biol. Toxicol..

[7] Hsu J.M. (1981). Lead toxicity as related to glutathione metabolism. J. Nutr..

[8] Winston D.W., Giulio R.T. (1991). Prooxidant and antioxidant mechanisms in aquatic organisms. Aquat. Toxicol..

[9] Xing J.Z., Zheu L., Jackson J.A., Gabos S., Sun X.J., Wang X.B. (2005). Dynamic monitoring of cytotoxicity on microelectronic sensors. Chem. Res. Toxicol..

[10] Schwartz R.A. (1997). Arsenic and the skin. Int. J. Dermatol..

[11] Guo T.L., Miller M.A., Shapiro I.M., Shenker B.J. (1989). Mercuric chloride induces apoptosis in human T lymphocytes: Evidence of mitochondrial dysfunction. Toxicol. Appl. Pharm..

[12] Flora S., Mittal M., Metha A. (2008). Heavy metal induced oxidative stress and its possible reversal by chelation therapy. Indian J. Med. Res..

[13] Wang G., Fowler B.A. (2008). Roles of biomarkers in evaluating interactions among mixtures of lead, cadmium and arsenic. Toxicol. Appl. Pharmacol..

[14] Pu Y., Hour T., Chen J., Huang C., Guan J., Lu S. (2002). Cytotoxicity of arsenic to transitional carcinoma cells. Urology.

[15] Lee T.C., Wei M.L., Chang W.J., Ho I.C., Lo J.F., Jan K.Y., Huang H. (1989). Elevation of glutathione levels and glutathione S-transferase activity in arsenic resistant Chinese hamster ovary cells. In Vitro Cell. Dev. Biol..

[16] Yang J.M., Arnush M., Chen Q.Y., Wu X.D., Pang B., Jiang X.Z. (2003). Cadmium induced damage to primary cultures of rat Leydig cells. Reprod. Toxicol..

[17] Masella R., di Benedetto R., Varì R., Filesi C., Giovannini C. (2005). Novel mechanisms of natural antioxidant compounds in biological system: Involvement of glutathione and glutathione-related enzymes. J. Nutr. Biochem..

[18] Stacchiotti A., Lavazza A., Rezzani R., Borsani E., Rodella L.F., Bianchi R. (2009). Mercuric chloride-induced alterations in stress protein distribution in rat kidney. Histol. Histopathol..

[19] Geier D.A., King P.G., Geier M.R. (2009). Mitochondrial dysfunction, impaired oxidative-reduction activity, degeneration, and cell death in human neuronal and fetal cells induced by low-level exposure to Thimerosal and other metal compounds. Toxicol. Environ. Chem..

